# Identification of Novel Variant of EML4-ALK Fusion Gene in NSCLC: Potential Benefits of the RT-PCR Method

**Published:** 2012-03

**Authors:** Martin K. H. Maus, Craig Stephens, Gary Zeger, Peter P. Grimminger, Eric Huang

**Affiliations:** 1 *Response Genetics, Inc., Los Angeles, CA, USA;*; 2 *Department of General, Visceral and Tumor Surgery, University of Cologne, GERMANY;*; 3 *Department of Pathology, Keck School of medicine, University of Southern California, USA*

**Keywords:** EML4-ALK rearrangement, EML4-ALK variants, NSCLC, ALK-Inhibitors

## Abstract

**Background:**

The discovery of the transforming fusion gene of the anaplastic lymphoma kinase (ALK) with the echinoderm microtubule-associated protein like 4 (EML4) as an oncogene in 2007 has led to its validation as a clinical target in NSCLC patients in a short period of time. The inhibition of the anaplastic lymphoma receptor tyrosine kinase has demonstrated to prolong progression-free survival compared to the standard of care chemotherapy in patients with advanced NSCLC that are ALK positive. However, the clinical implications of the 15 different variants of the EML4-ALK transforming gene described so far are currently not defined. Here we present a novel variant of the EML4-ALK fusion gene which we named variant 3c.

**Methods:**

RNA extracted from formalin fixed paraffin embedded (FFPE) specimens from patients with advanced and metastatic NSCLC was amplified, using primers and probes designed to detect specific EML4-ALK fusion gene fragments. Gel electrophoresis showed a different band for the new variant 3c compared to the known bands of positive cell lines for variant 3a and 3b. These findings were further investigated by dye-terminator Sequencing and FISH.

**Results:**

The novel variant, detected in two NSCLC specimens, is longer than v3a and shorter than v3b, representing an 18 base pair insertion of intron 19 of ALK between exon 6 of EML4 and exon 20 of ALK. All of the two samples showed exactly the same sequencing result. One of the samples was negative for FISH break apart testing and the other one showed a positive result, defined by ≥15% split nuclei as indicative of an ALK rearrangement.

**Conclusions:**

Compared to FISH technology, RT-PCR enables the detection of different isoforms of the EML4-ALK transforming gene, which can be validated by sequencing. Only one out of two samples that were positive for the new variant by RT-PCR could be confirmed by FISH. The clinical significance of the different variants, notably to resistance and response to ALK-Inhibitors and the concordance and sensitivity of FISH and RT-PCR should be subject to further investigations.

## INTRODUCTION

A small subset of 2-7% of patients with non-small cell lung cancer (NSCLC) share a rearrangement of the EML4 and ALK genes, both located on the short arm of chromosome 2 (1). Lung cancers that harbor this mutation derive oncogenicity from the ALK tyrosine kinase activity and share common biological and pathological features (2). The fusion of EML4 and ALK leads to constitutive activation of interconnected and overlapping pathways including the Ras-extracellular signal-regulated kinase (ERK) pathway, the Janus kinase 3 (JAK3) pathway and the phosphatidylinositol 3-kinase (PI3K)-Akt pathway which consecutively regulate transcription factors and genes involved in cell-cycle progression, proliferation and survival (3).

Patients with ALK positive lung cancers tend to be younger, with little or no exposure to tobacco and have mostly adenocarcinomas (4).

Since the ALK-tyrosine kinase activity is necessary for its oncogenic potential, a small molecule Inhibitor of the ALK-kinase was developed and assessed. In the phase I trial of PF-02341066, 82 patients with ALK positive NSCLC have shown a 57% overall radiographic response, according to the RECIST criteria, to ALK Inhibition by crizotinib. One of the key eligibility requirements for this study was ALK positivity on fluorescence in situ hybridization (FISH) with ≥15 % split nuclei as indicative of an ALK rearrangement.

Reverse-transcriptase-polymerase-chain-reaction (RT-PCR) was only performed retrospectively on a subgroup of FFPE tumor samples and with assays for ALK exon 20 and EML4-ALK exons 6, 13 and 18 (4). In contrast to FISH, which detects the break apart of the ALK gene in the 2p23 region regardless of what the inserted sequence or gene partner might be, RT-PCR technology enables the distinction between the different variants and fusion gene partners of the ALK gene by designing specific assays.

There are 15 different EML4-ALK fusion transcript variants identified to date, not including the rearrangement between ALK and other genes like TGF (chromosome 3) or KIF5B (chromosome 10), which both were identified as ALK-fusion partners from NSCLC specimens and are estimated to make up to 20% of the positive cases.^2^ The known variants 1, 2, 3a, 3b, 4, “4”, 5a, 5b, “5”, 6, 7, 8a, 8b, E17; ins68A20 and E20; ins18A20 include exon 2, 6, 13, 14, 15, 16, 17 and 20 of EML4 and exon 20 of ALK (5, 6). Variant 1 (33%), 3a/b (29%) and 2 (9%) seem to be the most frequent variants, the detection of the other variants is rare (2).

The clinical significance of these different variants in terms of a potential gradational response and implications on drug-resistance is currently unclear and remains subject to further investigations. Also, the sensitivity and specificity to quantitatively detect different variants and the concordance between FISH and RT-PCR needs to be further elucidated. However, here we present a novel variant of the EML4-ALK fusion gene.

## MATERIALS AND METHODS

**Figure 1:**
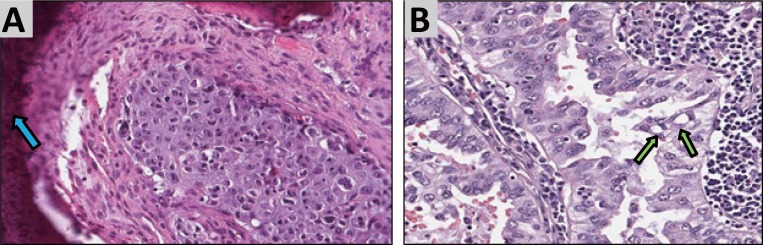
H&E slides from the two patients, that were tested positive for EML4-ALK Variant 3c. Image A shows poorly differenciated squamous cell carcinoma including bone (blue arrow) from a patient with history of squamous cell lung cancer. Image B shows moderately differenciated adenocarcinoma of a primary lung cancer. The green arrows indicate scattered signet ring cells.

Two formalin fixed paraffin embedded (FFPE) NSCLC specimens were tested positive for the new EML4-ALK fusion gene variant 3c by RT-PCR. The patients concerned were both male, 48 and 53 years old and had locally advanced and metastatic NSCLC. The histopathological reports showed moderately differentiated adenocarcinoma from a right upper lobe pneumonectomy in the first case and metastatic poorly differentiated squamous cell carcinoma from a left femoral head consistent with a lung primary for the other case (Fig. 1). A hematoxylin and eosin (H & E) stained section of each patients formalin-fixed paraffin embedded tumor specimen was evaluated by a board certified pathologist for tumor content.

Adjacent sections of the tumor were sectioned and stained with nuclear fast red (NFR) for visualization for microdissection. After microdissection of ≈40 mm^2^ tumor cells from 10 micron nuclear fast red (NFR) stained slides, RNA was extracted by the Response Genetics Inc. patented method. After isolation and lysis of the tumor cells, RNA and DNA were isolated separately from the specimen. The RNA was then reverse-transcribed to cDNA followed by RT-PCR analysis, using primers and probes designed to detect specific EML4-ALK fusion gene fragments with a maximum amplicon of 170 bases. The different assays are capable to detect and identify each of the variants 1, 2, 3a, 3b, 4, 5a and 7.

The primers and probes used to detect Variant 3 were 5΄-GCATAAAGATGTCATCATCAACCA-3΄ for the forward primer, 5΄-TGAGTACAAGCTGAGCAAGCTCCGC-3΄ for the reverse primer and 5΄-ACCAGGAGCTGCAAGCCATGC-3΄ for the probe. PCR cycling setting was 1 cycle for 15sec at 50°C, 10min at 95°C, 15sec at 95°C and 42 cycles for 1min at 62°C.

After hybridization and amplification of the specific template the finding was confirmed by agarose gel electrophoresis according to the length of the amplicon compared to positive-controls. For the detection of variant 3, two PCR products of 139 base pairs (corresponding to variant 3b) and 106 base pairs (corresponding to variant 3a), representing the linking of exon 6 of EML4 and exon 20 of ALK, translate to a specific band in gel electrophoresis which enables their differentiation. Slightly discrepant templates to the known sequence of variant 3a or 3b in terms of the number of base pairs inserted between the EML4 and ALK gene were also amplified in the PCR process because the primers are outside this region.

In this case, gel electrophoresis showed a different band compared to the bands of variant 3a and 3b. Those findings have been further investigated and confirmed by dye-terminator sequencing. A reaction cleanup was performed with Edge Biosystems filtration cartridges #42453 to purify sequencing reaction products after completion of PCR. After centrifugation of the cartridge and the sequencing reaction the sample was reconstituted in 20 μl of HiDi^TM^ Formamide, ABI catalog #4440753. Prior to loading to the ABI genetic analyzer 3130, the samples were denaturized for 2 min at 95°C. After that the fragments were separated according to their size by gel electrophoresis and the DNA bands were visualized with UV light leading to the sequence showed in the chromogram and Fig. 5.

In addition to that, fluorescence in situ hybridization (FISH) was performed using the Vysis ALK Break Apart FISH Probe Kit containing the LSI ALK Dual Color Probe.

## RESULTS

**Figure 2:**
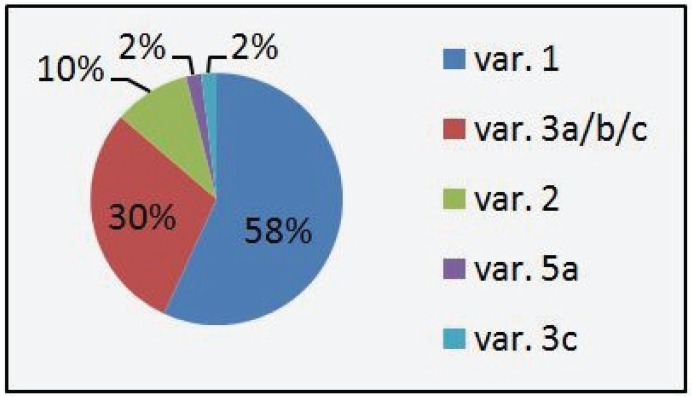
Frequency of EML4-ALK variants from the Response Genetics Inc. dataset 2010-2011.

**Figure 3:**
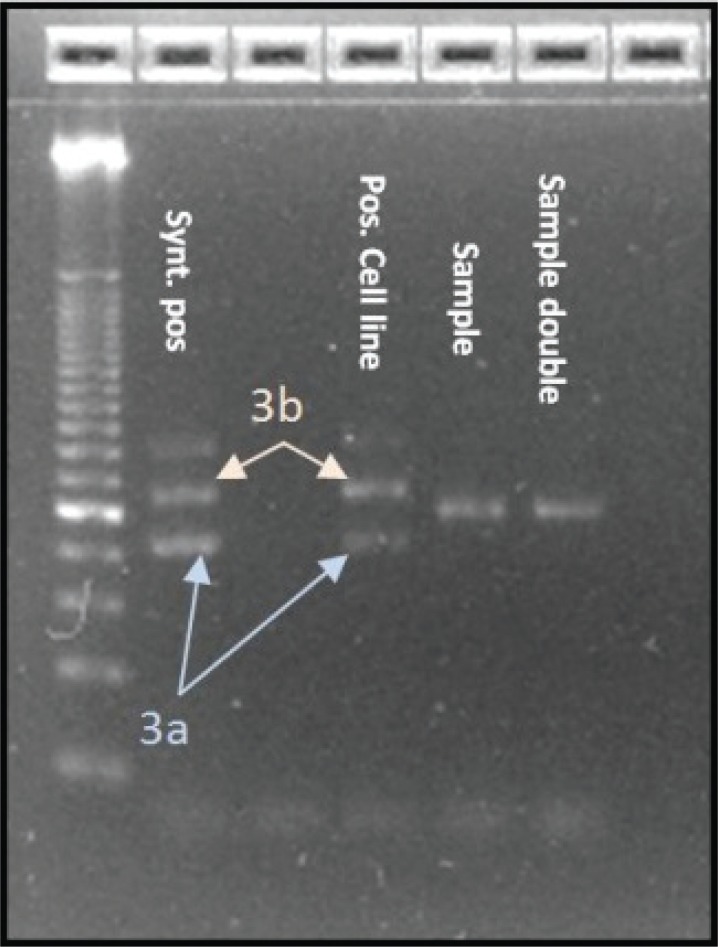
Image of Gel-Electrophoresis showing the bands of variant 3a/b and the band of variant 3c (sample & sample double) between them.

Over the last two years we detected 147 positive results (3%) out of 5000 NSCLC samples that were screened for EML4-ALK translocation variants 1, 2, 3a, 3b, 4, 5a, 6 and 7 by RT-PCR. Variant 1 was most frequent with 58% of the cases, followed by variants 3a/b/c with 30% and variant 2 with 10%. The newly indentified variant 3c as well as variant 5a represented 2% of the cases. Variant 4 and 7 were not detected in the collective. Figure 2 shows the distribution of the different EML4-ALK variants in our collective. None of the EML4-ALK positive samples, including variant 3c, showed concurrent KRAS or EGFR Mutations. The new variant of the EML4-ALK fusion gene that we identified shows similar features to variant 3a and 3b and therefore was named variant 3c. The band in agarose gel electrophoresis from the according samples, being in between the bands of positive cell lines and synthetic positive controls for variants 3a and 3b indicated a fragment which was longer than variant 3a but shorter than variant 3b (Figure 3). We then performed dye-terminator sequencing and found a chromogram which showed the sequence of an 18 base pair Insertion (5΄-CTGACCACCCACCTGCAG-3΄) of ALK intron 19 between EML4 exon 6 and ALK exon 20 (Figure 5). Variant 3a consists of a fusion between EML4 exon 6 and ALK exon 20 without any intronic sequence and variant 3b shows an Insertion of 33 base pairs (5΄-CAAAAATGTCAACTCGCGAAAAAAACAGCCAAG-3΄) similar to Variant 3c, with the difference that the v3b sequence derives from intron 6 of EML and the v3c sequence derives from intron 19 of ALK. Interestingly the inserted sequence is identical to a portion of the insertion in variant 5b between EML4 Exon2 and ALK exon 20 and can also be detected in Variant 8a and 8b.

**Figure 4:**
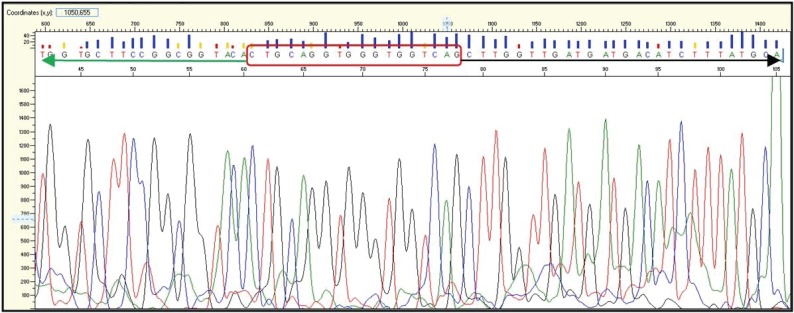
Chromogram of the sequence of variant 3c containing the 18 base pair insertion of Intron 19 of ALK (red box) and parts of Exon 6 of EML4 (black arrow) and Exon 20 of ALK (green arrow).

The whole test procedure was repeated and showed identical results. Both cases of variant 3c showed exactly the same sequence whose complimentary sequence is represented in the chromogram below (Figure 4).

**Figure 5:**
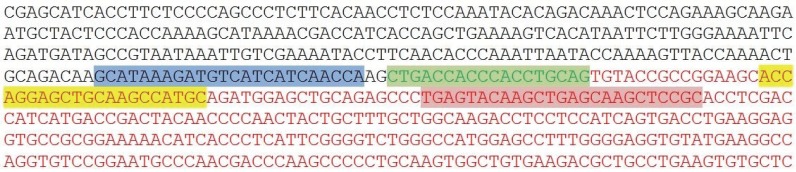
Sequence of the new variant 3c, showing the forward primer (blue), the intron 19 insertion (green), the probe (yellow) and the reverse primer (red). The EML4 gene is represented by black letters, the ALK gene by red letters. This seyuence is complimentary to the sequnce shown in the chromogram above (Fig. 3).

**Figure 6:**
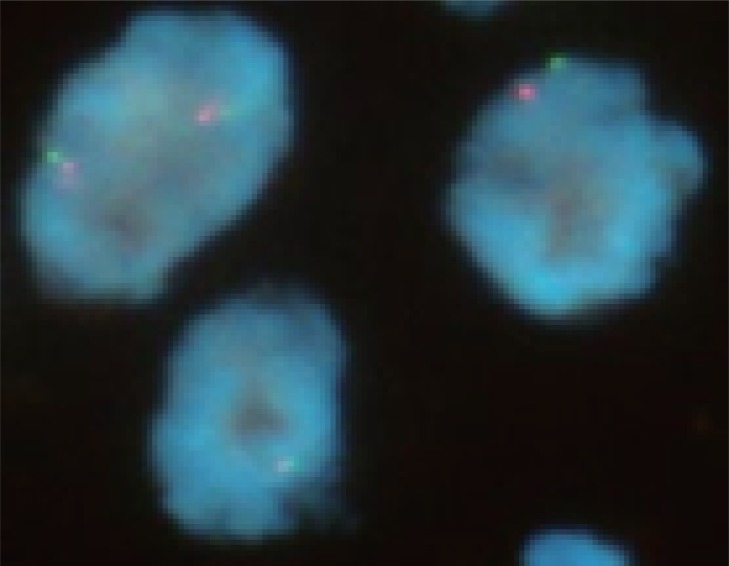
FISH Image showing two negative nuclei and one positive split nucleus (upper right).

In FISH testing one of the two cases was positive with 20 % split nuclei and the other negative with 13% split nuclei (Figure 6), setting the threshold for a positive result at ≥15 % split nuclei following the specifications from the clinical trial of crizotinib (PF-02341066), as indicative of an ALK rearrangement.

## DISCUSSION

In conclusion, we present a new variant of the EML4-ALK fusion gene, which shows a sequence of an 18 base pair insertion (5΄-CTGACCACCCACCTGCAG-3΄) of ALK intron 19 between EML4 exon 6 and ALK exon 20. The new variant contains the complete tyrosine kinase domain of ALK exon 20 as well as the amino-terminal coiled-coil domain within EML4 and therefore is likely to show activation of EML4-ALK (7). The fusion partner (EML4) in the translated fusion protein contains an oligomerization domain that mediates constitutive dimerization which leads to autophosphorylation and subsequent constitutive activation of the ALK kinase in the absence of the ligand (9).

The biology of variant 3c constitutes most likely a inversion on chromosome 2p like all other known variants of EML4-ALK so far. The different morphology of the variants originates from the variable truncation of the EML4 gene and intronic sequences belonging to either the EML4 or ALK gene. All variants 3 consist of a recombination of exon 6 of EML-4 and exon 20 of ALK but between them variant 3a shows no intronic sequence, variant 3b shows 33 base pairs from intron 6 of EML4 and variant 3c shows 18 base pairs from intron 19 of ALK. The sequence from variant 3c (5΄-CTGACCACCCACCTGCAG-3΄) could also be identified to be part of the intronic sequence of variant 5b, 8a and 8b, suggesting that the fusion of EML-4 to ALK occurred in different regions of intron 19 of ALK. Our RT-PCR results have been confirmed by dye-terminator sequencing and two cases of variant 3c have been identified to date.

The frequency of the different variants from our collective confirm other datasets, taken into account that our RT-PCR assays were specifically designed to detect variants 1, 2, 3a, 3b, 4, 5a, 6 and 7 not including variants “4”, 5b, “5”, 8a, 8b, E17;ins68A20, E20;ins18A20 and other fusion gene partners for ALK which also feature the amino-terminal coiled-coil domain, like TGF or KIF5B.

That leads to the question how meaningful it is to compare FISH and RT-PCR technology in a setting where FISH will show positive results regardless of the actual fusion gene partner and morphology of the rearrangement and RT-PCR constitutionally can only show positive results if a corresponding assay is covered by the test. In the clinical trial of crizotinib (PF-02341066) for example, only exon 6, 13 and 18 of EML4 exclusive of other potential variants or fusion gene partner was included in the RT-PCR test. Even though these exons constitute the most common fusion gene variants some variants and gene fusion partners can only be missed by RT-PCR in this setting. In addition to that, it is difficult to assess the specificity of the Vysis ALK Break Apart FISH Probe Kit, as it might also detect a break apart of the ALK gene independently from an activation of the ALK kinase, for example a fragmentation due to the preparation process and heating. All that could explain the low concordance of the two technologies and the higher sensitivity of FISH as described in this setting (4).

However, the most common variants so far identified are variant 1, 3 and 2 in this descending order and our group is currently working on a study comparing positive results preselected by RT-PCR to subsequent FISH testing. Preliminary data in this regard suggests that only about 50% of the PCR positive results can be confirmed by FISH, using the Vysis ALK Break Apart FISH Probe Kit and defining ≥15% split nuclei as indicative of an ALK rearrangement, indicating a higher sensitivity of RT-PCR compared to FISH. The determination and adjusting of the threshold for FISH positivity, which varies in different studies, is also strongly influencing the concordance between FISH and RT-PCR, which raises the question why 15% have been defined as a cutoff in the crizotinib trial. As the drug is designed to specifically inhibit the ALK kinase activity in every single positive tumor cell, what would be the correlation of the semi-quantitative evaluation of ALK positivity by counting split nuclei to the clinical response rate and would less but still positive tumor populations still benefit from the drug.

Backing the observations in our working group, it would be interesting to see how patients that are positive for RT-PCR but negative for FISH would respond to crizotinib. In addition to that, it could be possible that the degree of positivity for an ALK rearrangement in a patient varies over time and so could responsiveness.

The whole discussion about concordance between the FISH and RT-PCR method is also hindered by the fact that overall only few positive samples were available (2-7% of NSCLC patients). The benefit of RT-PCR to detect specific variants that are identified is in opposition to the benefit of FISH to show positive results even when we are dealing with new fusion gene partners, not detected so far.

Concerning the new variant 3c, only one out of two positive RT-PCR results could be confirmed by FISH. It remains unclear for now if the gradual response according to the RECIST criteria correlates within the variants and very little is known about the general clinical significance of the different variants and fusion gene partners. Another area of interest is how resistance mechanisms to crizotinib can be explained, observant that some ALK positive patients do not have a response to crizotinib (8). De novo mutated ALK genes could potentially explain some cases of developed resistance but the role of the different variants in this setting needs to be better understood too. The potential benefit of the RT-PCR method is the ability to detect and distinguish between those variants.

## References

[1] Soda M, Choi YL, Enomoto M (2007). Identification of the transforming EML4-ALK fusion gene in non-small-cell lung cancer. Nature.

[2] Sasaaki S, Rodig S, Chirieac LR, Jänne PA (2010). The biology and treatment of EML4-ALK non-small cell lung cancer. European Journal of Cancer.

[3] Takezawa K, Okamoto I, Nishio K, Jänne PA (2011). Role of ERK-BIM and STAT3-Survivin Signaling Pathways in ALK Inhibitor–Induced Apoptosis in EML4-ALK–Positive Lung Cancer. Clinical Cancer Research.

[4] Kwak EL, Bang YJ, Camidge R (2010). Anaplastic Lymphoma Kinase Inhibition in Non-Small-Cell Lung Cancer. The New England Journal of Medicine.

[5] Sanders HR, Li HR, Bruey J-M (2011). Exon scanning by reverse transcriptase-polymerase chain reaction for detection of known and novel EML4-ALK fusion variants in non-small cell lung cancer. Cancer Genetics.

[6] Choi YL, Takeuchi K, Soda M (2008). Identification of Novel Isoforms of the EML4-ALK Transforming Gene in Non-Small Cell Lung Cancer. Cancer Research.

[7] Zhang X, Zhang S, Yang X (2009). Fusion of EML4 and ALK is associated with development of lung adenocarcinomas lacking EGFR and KRAS mutations and is correlated with ALK expression. Molecular Cancer.

[8] Takaaki Sasaki T, Koivunen J, Ogino A (2011). A Novel ALK Secondary Mutation and EGFR Signaling Cause Resistance to ALK Kinase Inhibitors. Cancer Research.

[9] Solomon B, Varella-Garcia M, Camidge D (2009). ALK Gene Rearrangements A new therapeutic target in an molecularly defined subset of non-small cell lung cancer. Journal of Thoracic Oncology.

